# Author response to: Comment on: Surgeon age in relation to patients’ long-term survival after gastrectomy for gastric adenocarcinoma: nationwide population-based cohort study

**DOI:** 10.1093/bjsopen/zrae101

**Published:** 2024-09-11

**Authors:** Wilhelm Leijonmarck, Jesper Lagergren

**Affiliations:** Department of Molecular Medicine and Surgery, Karolinska Institutet and Karolinska University Hospital, Stockholm, Sweden; Department of Molecular Medicine and Surgery, Karolinska Institutet and Karolinska University Hospital, Stockholm, Sweden; School of Cancer and Pharmacological Sciences, King’s College London, London, UK

We appreciate the comments by DiaS *et al*.^1^ and their interest in our study on the association between surgeon age and long-term survival after gastrectomy for gastric adenocarcinoma^2^. They raise concerns about the predominance of low surgeon and hospital volumes of gastrectomy, the study design, and the fact that surgical experience and skill are not fully captured in the analyses.

The relatively low surgeon volume of gastrectomy for gastric adenocarcinoma is mainly due to the low incidence in Sweden. Centralization has reduced short-term mortality, but it remains unclear whether higher surgeon and hospital volumes improve long-term survival in low-volume Western countries like Sweden. In our study, we considered surgeon volume as a mediator. The point estimates did not change after inclusion of surgeon volume in a mechanistic model, suggesting that surgeon volume may be of less importance in this cohort. The results may reflect the association between surgeon age and long-term mortality after gastrectomy for gastric adenocarcinoma in settings with relatively low surgeon and hospital volumes, and might not be generalizable to other settings. We welcome the suggestion by DiaS *et al*.^1^ for similar studies in countries with higher surgeon and hospital volumes.

Surgeon proficiency in gastrectomy could additionally be influenced by experience gained from other major gastrointestinal procedures. In our study, we evaluated lymph node yield, resection margin status, postoperative complications, and surgeon volume, but there may be other aspects of surgical skills that were not possible to account for.

Regarding selection bias, a main strength of our study is the population-based design, which enabled an unselected cohort with 98% completeness of patients who underwent gastrectomy for gastric adenocarcinoma in Sweden between 2006 and 2015. Thus, selection bias should be minimal.
